# Immune Landscape and Tumour Heterogeneity in Ovarian Cancer: Insights From Single‐Cell RNA Sequencing

**DOI:** 10.1111/jcmm.71235

**Published:** 2026-06-11

**Authors:** Sihan Chen, Ghada Moh. Samir Elhessewi, Helen Cai, Wedad M. Alawad, Manoj Kumar Mishra, Usamah Sayed

**Affiliations:** ^1^ ENT Clinical Fellow University Hospitals Bristol and Weston, Bristol Royal Infirmary Bristol UK; ^2^ Department of Health Sciences, College of Health and Rehabilitation Sciences Princess Nourah bint Abdulrahman University Riyadh Saudi Arabia; ^3^ Middlesex Business School Middlesex University London UK; ^4^ Department of Information Technology, College of Computer Qassim University Buraydah Saudi Arabia; ^5^ Salale University Fitche Ethiopia; ^6^ Faculty of Allied Medical Sciences, Hourani Center for Applied Scientific Research Al‐Ahliyya Amman University Amman Jordan

**Keywords:** Bioinformatics, immune heterogeneity, immunotherapy, ovarian cancer, single‐cell RNA sequencing, tumour microenvironment

## Abstract

Tumour heterogeneity is a key factor in cancer progression, with immune responses within the tumour microenvironment (TME) contributing significantly to treatment resistance and immunotherapy outcomes. Recent advances in single‐cell RNA sequencing (scRNA‐seq) have provided unprecedented insights into the diverse immune cell populations infiltrating tumours, including both innate immune cells like dendritic cells, neutrophils, macrophages, and natural killer (NK) cells, as well as adaptive immune cells such as T lymphocytes. The immune landscape of tumours is complex and dynamic, characterised by a mixture of activated and suppressed immune states that evolve over time. The degree of immune cell infiltration varies among tumour types and disease stages, influencing tumour response to therapies. For example, ovarian cancer typically exhibits weaker immune infiltration compared to cancers like melanoma and non‐small cell lung cancer. Increased CD8+ T cell infiltration is generally associated with favourable prognosis, while elevated regulatory T cells (Tregs) can suppress anti‐tumour immune responses. The use of scRNA‐seq has enabled detailed profiling of immune cells, revealing the roles of exhausted T cells and immunosuppressive macrophages in the TME. This high resolution approach facilitates the identification of distinct immune cell subsets and their functional states, providing a platform for developing more targeted, personalised immunotherapies. The findings offer promising avenues for improving clinical outcomes in ovarian cancer and other malignancies through refined immune profiling.

## 1 Introduction

Ovarian cancer (OC) is a tumour type that shows promise for personalised medicine. Despite advances in therapeutic strategies, survival rates remain suboptimal, largely due to late‐stage diagnosis and extensive intratumoral heterogeneity [1, 2, 3]. Recent advances in diagnosis and treatment have improved survival rates, from about 37% to nearly 50% [4]. This improvement reflects better medical and scientific understanding of the disease, though much remains to be understood. The causes of ovarian cancer are complex, involving genetics, environment, and lifestyle factors [5]. As research into tumour biology progresses, it is becoming clear that both cancer cell characteristics and the tumour microenvironment (TME) play key roles in how the disease develops and responds to treatments. These elements are now considered critical for developing individualised treatment strategies [6].

The TME is a varied and active environment made up of tumour cells and surrounding components, both cellular and non‐cellular. The TME includes immune‐infiltrating cells (IICs), stromal cells, endothelial cells (ECs), and the extracellular matrix, along with various signalling molecules [7, 8]. Together, these elements form a network that can influence how a tumour grows, invades surrounding tissue, metastasizes, and responds to therapy. This network is essential for tumour proliferation, immune system evasion, blood vessel formation (angiogenesis), and resistance to treatments like chemotherapy and immunotherapy [8, 9]. Because of the complexity of the TME, research is increasingly focusing not just on the tumour cells themselves but on how they interact with the surrounding environment. It's becoming clear that understanding these interactions is crucial for effective treatment strategies. Immune cells are often found within the stromal compartment of the TME. “Immune‐infiltrating cells” refers to those that enter the tumour tissue, while immune cells in the matrix are more about where they are located in the stroma. In practice, the line between these categories is often blurred, and much of the focus is on how immune cells interact with other parts of the TME [10]. For the sake of this discussion, we will treat all immune cells within the TME as part of the stromal compartment. Stromal cells, both immune and non‐immune, such as fibroblasts, mesenchymal cells, and endothelial cells, are a major research focus, especially in the context of tumour immunotherapy [11]. The behaviour and characteristics of these stromal components are shaped by a mix of internal and external factors. Despite much progress, the heterogeneity and variability within the TME still pose significant challenges for research, highlighting the need for new investigative tools. In ovarian cancer specifically, the peritoneal microenvironment and ascitic fluid create a unique immunosuppressive niche that further complicates treatment responses, highlighting the need for more precise investigative tools [6, 12] (Table 1).

**TABLE 1:** Tumour Microenvironment (TME) Components in ovarian cancer and their roles in tumour progression [11, 13].

Component	Description	Role in tumour progression	Challenges
Immune‐infiltrating cells (IICs)	Include dendritic cells, neutrophils, macrophages, and NK cells.	Play roles in immune surveillance and immune evasion.	Complexity in immune cell functions and interactions.
Stromal cells	Fibroblasts, mesenchymal cells, and endothelial cells.	Support tumour growth and metastasis; involved in angiogenesis.	Heterogeneity of stromal components complicates therapy.
Extracellular matrix (ECM)	A network of proteins and signalling molecules surrounding tumour cells.	Supports tumour growth and invasion, influences immune response.	Resistance to treatments like chemotherapy and immunotherapy.
Peritoneal microenvironment & ascitic fluid	Unique to ovarian cancer, contributing to immunosuppressive niche.	Adds complexity to treatment resistance and immune evasion.	Challenges in targeting immune suppression.

## 2 Recent Advancements in Genomic Technologies

Recent developments in genomic technologies have introduced powerful tools like bulk RNA sequencing, single‐cell DNA and RNA sequencing, single‐cell proteomics, and single‐cell metabolomics. These technologies allow for a much deeper, more detailed exploration of complex diseases such as cancer, opening up new possibilities for more precise and effective treatments. Of particular interest is how single‐cell approaches are helping to reveal the complex features of the TME in ovarian cancer. This review explores how single‐cell analysis is being used to study the stromal components of the TME in ovarian cancer, highlighting key findings and emerging trends in this fast‐moving field [6, 14].

## 3 Overview of scRNA‐Seq Technology

Directly sequencing RNA from single cells is technically challenging. To make single‐cell RNA sequencing (scRNA‐seq) possible, the RNA is first converted into complementary DNA (cDNA) and then amplified using methods like polymerase chain reaction (PCR) or in vitro transcription (IVT). 13Various iterations of scRNA‐seq have been developed to address these challenges, improving both performance and the range of potential applications. Each new version has its own strengths and focuses on different aspects of molecular detection. The typical scRNA‐seq workflow involves four main steps: isolating individual cells, converting RNA to cDNA, amplifying the cDNA, and then constructing a sequencing‐ready library [12, 15].

## 4 Single‐Cell Isolation Methods

There are several methods for isolating single cells, including manual selection, dilution, random seeding, laser capture microdissection, fluorescence‐activated cell sorting (FACS), and newer techniques like microfluidics or microplates. Among these, FACS is the most widely used due to its precision. While manual selection was common in earlier studies, it's inefficient and not scalable. Microfluidic systems, like Drop‐seq, are more recent and offer significant advantages by encapsulating single cells in droplets with primers, DNA barcodes, and molecular tags. This enhances throughput and allows for parallel processing of thousands of cells, which is particularly valuable for large‐scale transcriptomic studies [16, 17].

## 5 scRNA‐Seq Protocols

Various scRNA‐seq protocols have been developed, each with its strengths and limitations. The first major method, the Tang protocol, used micromanipulation for cell isolation, but it had low sensitivity and accuracy. Smart‐seq, which uses reverse transcriptase from Moloney murine leukaemia virus (MMLV), allows for full‐length cDNA generation and improves sensitivity and precision. Other methods, like STRT‐seq and STRT/C1‐seq, use unique molecular identifiers (UMIs) to improve transcript quantification but tend to favour the 5′ ends of transcripts. CEL‐seq and CEL‐seq2, which use in vitro transcription (IVT), are good for detecting the 3′ ends of transcripts but suffer from reduced accuracy and a strong 3′ bias. Drop‐seq is great for sequencing large numbers of cells but has lower sensitivity for low‐abundance transcripts [18, 19].

## 6 Reverse Transcription and cDNA Amplification

**FIGURE 1:**
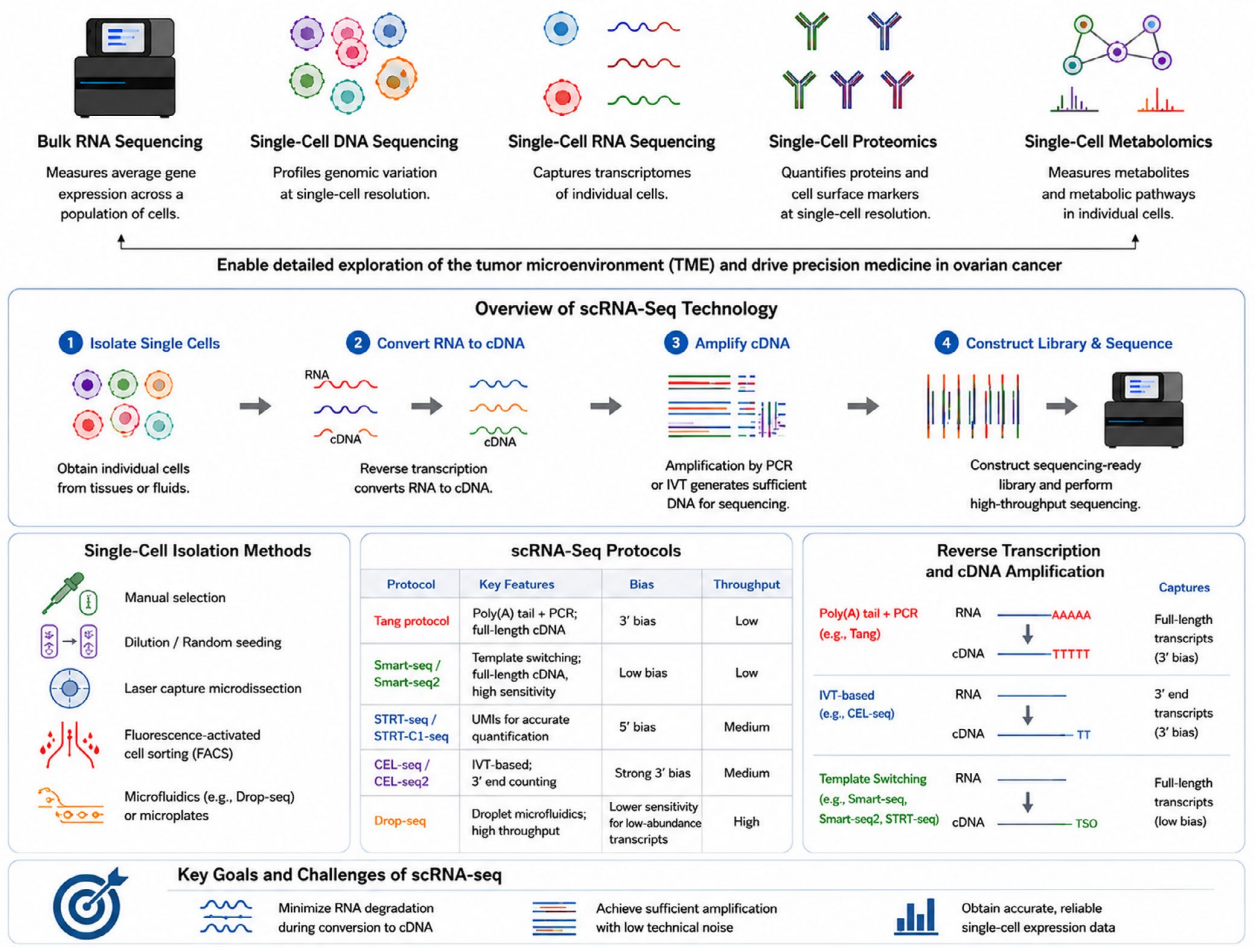
Overview of recent advancements in genomic technologies, focusing on single‐cell RNA sequencing (scRNA‐seq) in ovarian cancer research. This figure illustrates the key steps in the scRNA‐seq workflow, including cell isolation, RNA conversion to cDNA, amplification, and sequencing. It also highlights various single‐cell isolation methods, protocols, and reverse transcription techniques, along with their respective biases and throughput.

Reverse transcription and cDNA amplification are key steps that impact the accuracy and sensitivity of scRNA‐seq. Most methods use oligo (dT) primers to reverse‐transcribe polyadenylated transcripts, but this excludes non‐coding RNAs like long non‐coding RNAs (lncRNAs) and circular RNAs. Depending on the approach, scRNA‐seq can be divided into three main types: those that use poly(A) tail addition with PCR, IVT‐based methods, and template‐switching techniques. In the poly(A) tail method, like the Tang protocol, synthetic poly(A) sequences are added to the 3′ end of RNA to facilitate full‐length transcript amplification. While this method helps detect previously uncharacterised transcripts, it has a 3′ bias and lower efficiency, which reduces sensitivity. IVT methods, which use primers with a T7 promoter, can also introduce substantial 3′ bias due to their directional nature. Template‐switching techniques, used in Smart‐seq, Smart‐seq2, and STRT‐seq, are designed to reduce 3′ bias by adding non‐templated nucleotides at the 3′ end of cDNA. This process captures full‐length transcripts and improves the coverage of the transcriptome [20, 21] (Figure 1).

## 7 Barcoding and UMIs in scRNA‐Seq

Before sequencing, individual cells are typically barcoded to ensure each one is uniquely identified. This method allows multiple samples to be processed together in a single sequencing run, preventing any mix‐up. It streamlines the workflow and reduces reagent use by pooling samples early on and amplifying them together. To reduce PCR bias, unique molecular identifiers (UMIs) short nucleotide sequences are added during reverse transcription. These UMIs help differentiate real transcripts from PCR duplicates, improving the accuracy of transcript quantification. Techniques like CEL‐seq, CEL‐seq2, STRT‐seq, STRT/C1‐seq, and Drop‐seq use UMIs to enhance reliability, with STRT‐seq using streptavidin‐coated beads to simplify library prep and quality control [12, 21, 22].

## 8 Data Analysis Challenges and Tools

Data analysis is a crucial part of scRNA‐seq due to the low RNA input from single cells. This small amount of RNA requires amplification, which can introduce variability and errors, such as “dropout” events where certain transcripts aren't detected even though they're present. This results in a lot of zero values in gene expression data. To address this, various specialised normalisation and analysis tools have been developed. Some of the most widely used platforms include Seurat, Monocle, Scanpy, and Linnorm, which handle tasks like quality control, normalisation, dimensionality reduction, clustering, and differential expression analysis. Seurat is a comprehensive tool for analysing transcriptomic variation across cells, helping classify subgroups and identify marker genes. Monocle, on the other hand, focuses on arranging cells along differentiation trajectories, providing insights into gene regulation over time. Scanpy, a Python‐based tool, is great for handling large datasets and offers functions for visualisation, trajectory analysis, and gene regulatory network construction. Additional tools like Linnorm, SCnorm, scran, and DESeq provide more flexibility for different experimental setups [23, 24, 25, 26].

## 9 Commercial Platforms and Innovations

On the commercial side, leading platforms for scRNA‐seq include 10X Genomics and BD Rhapsody, both of which offer integrated workflows. These systems can process cells, build libraries, sequence, and even carry out some preliminary analysis in under 30 min. Recent innovations in single‐cell transcriptomics have also led to the development of platforms that integrate scRNA‐seq with complementary technologies. One such innovation is CITE‐seq (Cellular Indexing of Transcriptomes and Epitopes by sequencing), which combines transcriptome analysis with cell surface protein profiling. CITE‐seq uses barcoded antibodies that bind to cell surface markers, allowing researchers to measure both mRNA expression and protein levels from the same cell. This method overcomes a major limitation of traditional scRNA‐seq, which typically doesn't provide protein‐level data. By capturing both transcriptome and protein data during sequencing, CITE‐seq enables a more comprehensive view of cellular function [27, 28].

## 10 CROP‐Seq: Integrating CRISPR With scRNA‐Seq

CROP‐seq (CRISPR droplet sequencing) is an innovative technique that combines genome editing with single‐cell RNA sequencing to functionally explore genes at a single‐cell level. This method uses guide RNAs (gRNAs) that are traceable in the transcriptomic data, making it possible to link specific genetic alterations with changes in gene expression. The high‐throughput sequencing process, combined with a computational pipeline, helps to associate gRNA presence with gene expression shifts. This allows for a more precise dissection of regulatory networks and the diverse cellular responses that occur in different conditions, providing deeper insights into gene function and potential therapeutic targets [29, 30] (Table 2).

**TABLE 2:** Key single‐cell RNA sequencing (scRNA‐seq) technologies and protocols [31, 32].

Technology	Purpose	Strengths	Limitations
Bulk RNA sequencing	Analysing gene expression across bulk tissue samples.	Allows for broad profiling of gene expression.	Masks cellular diversity and heterogeneity.
scRNA‐seq	Sequencing RNA from individual cells.	High resolution, captures individual cell variability.	Technical challenges in RNA quality and amplification.
Drop‐seq	Uses microfluidic systems to isolate single cells.	High throughput, enables large‐scale transcriptomic studies.	Lower sensitivity for low‐abundance transcripts.
Smart‐seq	Uses reverse transcriptase for cDNA synthesis.	High sensitivity and precision, full‐length transcript coverage.	May have a bias toward 3′ ends of transcripts.

## 11 Tumour Heterogeneity and Progression Through scRNA‐Seq

Cancer heterogeneity is a hallmark of ovarian cancer and plays a critical role in treatment resistance and disease progression. While earlier studies relied on bulk sequencing approaches, these methods masked cellular diversity. It's a highly heterogeneous tumour, meaning it has many different cell types and genetic features even within a single tumour. This variability complicates treatments and makes it harder to predict how the disease will progress in each individual. scRNA‐seq has been a game changer, giving us a closer look at what's happening on the molecular level. It lets us look at individual cells, their characteristics, and how they interact with each other. Studies have shown that ovarian cancer is not just one disease but many different forms that act and respond to treatments in their own way [12]. scRNA‐seq studies have directly characterised intratumoral heterogeneity in ovarian cancer. For example, scRNA‐seq analyses of high‐grade serous ovarian cancer (HGSOC) have revealed distinct malignant cell subpopulations with varying transcriptional programs related to proliferation, metastasis, and drug resistance. These studies have identified epithelial‐to‐mesenchymal transition (EMT)‐like states and stem‐like populations that are strongly associated with poor prognosis and chemoresistance. Additionally, single‐cell profiling of primary and metastatic ovarian tumours has demonstrated significant transcriptional divergence, particularly in omental metastases, where tumour cells adapt to a lipid‐rich microenvironment. Importantly, ovarian cancer‐specific studies have shown that tumour evolution is not linear but rather [3, 33].

Single‐cell RNA sequencing (scRNA‐seq) takes this further by providing a detailed view of the molecular landscape at an unprecedented resolution. It is particularly useful for studying tumour evolution, from early stages to more advanced and metastatic forms [19]. For example, in pancreatic ductal adenocarcinoma (PDA), which has a long preclinical phase, scRNA‐seq of epithelial cells with pancreatic intraepithelial neoplasia (PanIN) has revealed transcriptional changes linked to cell proliferation, invasion, and metastasis. This approach not only helps identify the risk of malignant transformation early on but also informs more targeted therapeutic strategies. Additionally, scRNA‐seq is used to track the progression of diseases in animal models, from early stages like acinar‐to‐ductal metaplasia (ADM) to invasive cancer [34]. These studies often highlight distinct gene expression patterns and cell variability, leading to the identification of new molecular targets for treatment. Tumour metastasis, which is responsible for most cancer‐related deaths, involves the spread of cancer cells to distant organs. By comparing primary tumours with metastatic ones, scRNA‐seq has uncovered significant genetic and transcriptional differences [35].

## 12 scRNA‐Seq in Tumour Heterogeneity and Progression

Ovarian tumours show incredible diversity in terms of their genetic make‐up. This genetic variability can be huge, with some tumours showing widespread chromosomal instability or mutations in certain genes like TP53. These mutations are often linked to how the tumour evolves over time. The problem with this kind of diversity is that it makes the cancer harder to treat because no two parts of the tumour might be responding the same way to treatment. This variability is also reflected in the way the cancer cells behave at the transcriptional level. Through scRNA‐seq, we've learned that these tumours are made up of different subpopulations, each with distinct behaviours; some might be highly aggressive, others might be more resistant to treatments [3]. In addition to the genetic differences, ovarian cancer cells also exhibit transcriptional variation. These differences are essential in determining how these cells behave in the body and their ability to evade immune responses or resist chemotherapy. Understanding these variations gives researchers insight into why ovarian cancer is so difficult to treat effectively and why one treatment might work for one patient but not another. Through scRNA‐seq, we can finally get a better grasp on how these different cell types contribute to the overall behaviour of the tumour [6].

In ovarian cancer, particularly HGSOC, scRNA‐seq has been extensively applied to dissect transcriptional heterogeneity associated with tumour progression and metastatic dissemination. Recent single‐cell studies have demonstrated that ovarian tumours exhibit diverse malignant cell subpopulations characterised by distinct transcriptional programs linked to proliferation, epithelial–mesenchymal transition (EMT), and chemoresistance. Unlike genetically driven models such as VHL loss in renal cancer, ovarian cancer progression is more strongly associated with transcriptional plasticity and microenvironmental adaptation, especially within the peritoneal cavity [6, 33].

scRNA‐seq analyses of primary ovarian tumours and matched metastatic sites, such as the omentum, have revealed significant transcriptional reprogramming, including upregulation of pathways involved in lipid metabolism, extracellular matrix remodelling, and immune evasion. Key signalling molecules such as EGFR, SRC, and PI3K pathway components have also been identified as upregulated in aggressive ovarian cancer cell populations, suggesting their potential as therapeutic targets in combination treatment strategies. These findings are increasingly supported by functional validation in both in vitro organoid models and in vivo systems. Beyond metastatic progression, scRNA‐seq has significantly advanced the classification of ovarian cancer at the cellular level, enabling the identification of distinct malignant cell states, including stem‐like, proliferative, and mesenchymal‐like subpopulations. These cellular states are not fixed but exist along dynamic transcriptional trajectories, which can be reconstructed using pseudotime analysis to better understand tumour evolution and treatment resistance [6, 33].

Furthermore, the integration of scRNA‐seq with epigenetic and microenvironmental data has revealed that ovarian tumour heterogeneity is strongly influenced by stromal and immune interactions, rather than purely intrinsic genetic alterations. This contrasts with other malignancies such as glioblastoma or acute myeloid leukaemia, where lineage hierarchies are more clearly defined. In ovarian cancer, cellular identity appears to be highly context‐dependent, shaped by factors such as hypoxia, cytokine signalling, and interactions with cancer‐associated fibroblasts and immune cells. Tthese findings highlight that scRNA‐seq provides a powerful framework for understanding the complex and dynamic ecosystem of ovarian cancer, enabling more precise tumour classification and offering new opportunities for the development of targeted and personalised therapeutic strategie [6, 12, 33].

## 13 Epigenetic Regulation and Ovarian Cancer

**FIGURE 2:**
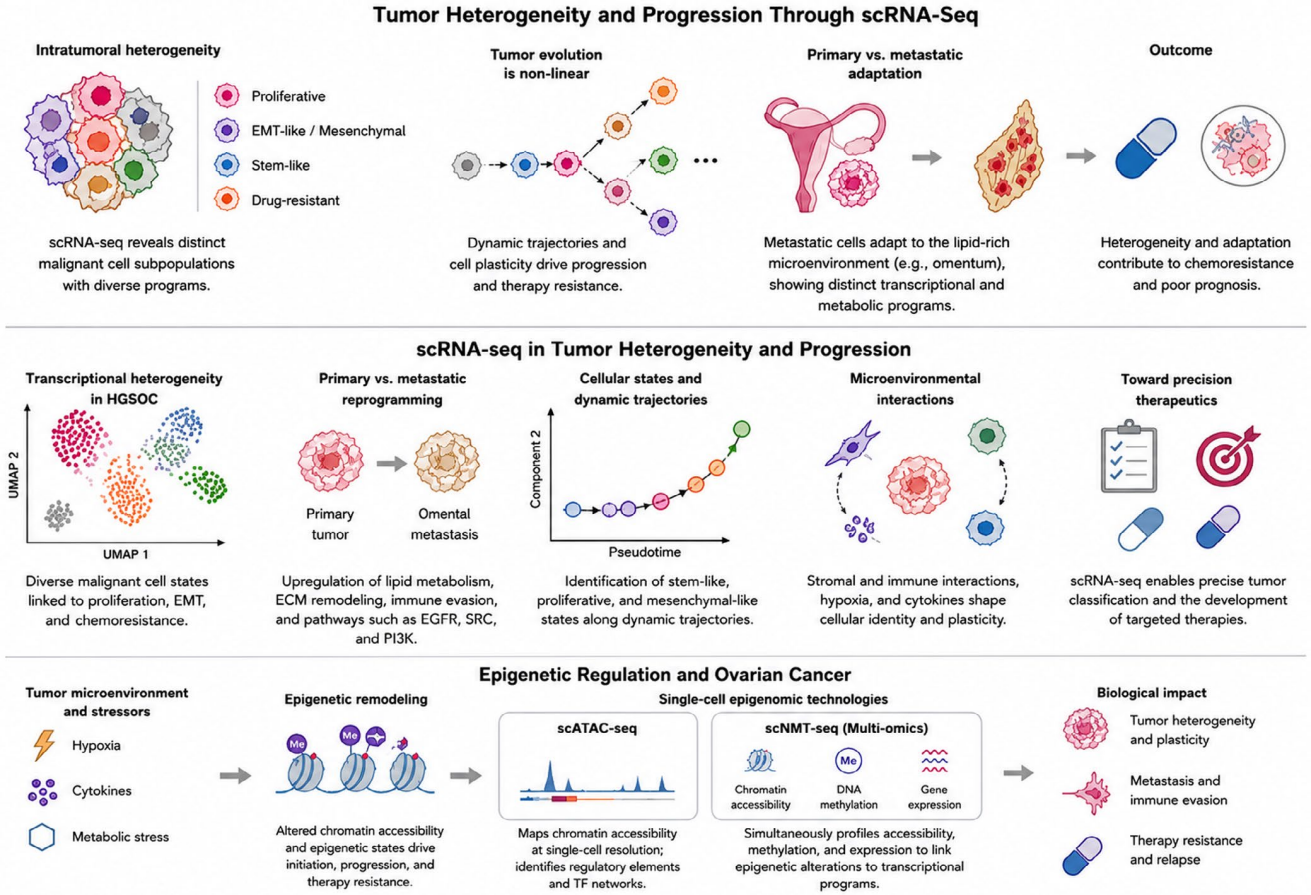
Schematic representation of tumour heterogeneity and progression in ovarian cancer through single‐cell RNA sequencing (scRNA‐seq). This figure illustrates the dynamic evolution of tumour cells, from intratumoral heterogeneity to the adaptation of metastatic cells in the lipid‐rich microenvironment. Key stages include transcriptional reprogramming, cellular state transitions, and the identification of stem‐like, proliferative, and mesenchymal‐like cell populations. The interplay between tumour microenvironment interactions and epigenetic remodelling is also highlighted, emphasising the potential of scRNA‐seq to guide precision therapies and inform targeted treatment strategies for ovarian cancer.

While genomic mutations have traditionally been the main focus in cancer research, recent work has emphasised the critical role of epigenetic regulation in driving tumour heterogeneity, particularly in ovarian cancer. In this disease, changes in chromatin structure and epigenetic state contribute to tumour initiation, progression, and therapy resistance. Environmental and metabolic stressors within the tumour microenvironment can alter chromatin organisation, enabling precancerous ovarian epithelial cells to transition toward malignancy or allowing established tumour cells to adapt and survive under treatment pressure [33]. In ovarian cancer research, integrating single‐cell RNA sequencing (scRNA‐seq) with epigenetic profiling has become an important strategy for dissecting tumour complexity. One widely used approach is single‐cell ATAC‐seq (scATAC‐seq), which maps chromatin accessibility at the level of individual ovarian cancer cells. This technique has revealed distinct regulatory landscapes across tumour subclones, highlighting key enhancer regions and transcription factor networks associated with proliferation, metastasis, and chemoresistance. More comprehensive multi omics methods, such as scNMT‐seq, further extend these insights by simultaneously measuring chromatin accessibility, DNA methylation, and gene expression within the same ovarian cancer cell. This integrated view allows researchers to directly link epigenetic alterations to transcriptional programs, offering a deeper understanding of how epigenomic remodelling drives cellular plasticity during ovarian cancer progression and treatment response [12] (Figure 2).

## 14 Cancer Stem Cells and Therapeutic Implications

Single‐cell transcriptomics has provided significant evidence supporting the cancer stem cell (CSC) model, which suggests that a subset of self‐renewing cells within a tumour is responsible for tumour heterogeneity, metastasis, and resistance to treatment. These CSCs contribute to the formation of various non‐cancerous cell types in the tumour microenvironment, making them key targets for therapeutic strategies. Technologies like single‐cell RNA sequencing (scRNA‐seq) have been instrumental in identifying these CSC populations and understanding the signalling pathways, such as Wnt and Notch, that regulate their behaviour [36]. By using models like 3D organoids and patient‐derived xenografts (PDX), studies have shown that inhibiting these key pathways can slow down CSC‐driven tumour growth. Additionally, pseudotemporal trajectory analysis of single‐cell data has highlighted how CSCs differentiate into various tumour subtypes over time. In the case of collecting duct renal cell carcinoma (CDRCC), scRNA‐seq revealed that CSCs play a pivotal role in tumour progression, with markers such as BIRC5 and CDKN3 being highly expressed, which are associated with poorer clinical outcomes. These findings suggest that targeting pathways specific to CSCs could offer a promising therapeutic approach [37].

## 15 Limitations and Advances in scRNA‐Seq

Despite its advantages, scRNA‐seq has some limitations. Technical challenges include biases in transcript capture (whether from the 3′ or 5′ end), limited sensitivity for low‐abundance transcripts, and sequencing depth constraints, all of which can lead to noise in the data. To address these, new techniques have been developed. For instance, Genotyping of Transcriptomes (GoT) integrates somatic mutation detection with transcriptomic profiling in thousands of individual tumour cells, overcoming issues related to low gene expression or transcripts that are far from capture sites [18, 19]. Tools like inferCNV also help detect copy number variations by comparing gene expression patterns between tumour cells and reference cells, enhancing the interpretation of large‐scale genomic events [23]. In summary, while challenges remain, scRNA‐seq has revolutionised the study of cancer heterogeneity. It offers a level of resolution that allows for a deeper understanding of tumour development, metastasis, treatment resistance, and epigenetic regulation. As the technology continues to improve and integrate with other omics platforms, it will likely play a key role in advancing personalised cancer therapies [12].

## 16 Immune Profiling and Tumour Immune Diversity With scRNA‐Seq

The immune system in ovarian cancer is incredibly complex. Some immune cells within the tumour seem to work in favour of the cancer, promoting growth and preventing effective immune responses. These cells include regulatory T cells (Tregs) and certain types of macrophages, which tend to suppress the immune system's ability to target cancer. On the other hand, there are also immune cells that fight against the tumour, like cytotoxic T cells. The balance between these different cell types plays a big role in how the disease progresses. Using scRNA‐seq, researchers can now identify immune cells at a much finer level than before and understand their specific roles in the tumour microenvironment [33].

One thing that's come through clearly in the recent research is that ovarian tumours create an environment that can turn immune cells into “friends” or “enemies” of the cancer. Some macrophages, for example, can be “reprogrammed” by the tumour to help it grow, while others might try to kill the cancer cells. Similarly, T cells, which are usually the body's main defence against cancer, can become “exhausted” in the tumour microenvironment and stop fighting. ScRNA‐seq has helped researchers track these immune cells in ways that weren't possible before. By identifying which immune cells are exhausted or dysfunctional, researchers hope to find ways to wake them up and get them back to work [6].

Tumour heterogeneity is a critical factor in cancer progression, and much of this variability stems from the diverse immune responses within the TME [8]. This immune diversity significantly influences how tumours evade treatment and their responsiveness to immunotherapies. Recent advancements in single‐cell RNA sequencing (scRNA‐seq) have allowed researchers to uncover a broad spectrum of immune cell infiltrates within tumours [19]. These infiltrates include innate immune cells such as dendritic cells, neutrophils, macrophages, and natural killer (NK) cells, as well as adaptive immune cells, including various subsets of T lymphocytes. The presence of both activated and suppressed immune cell states within the TME indicates a highly complex and dynamic immune landscape that likely evolves over time. Immune heterogeneity, however, is not merely defined by the types of immune cells present but also by their abundance and spatial distribution within the tumour. Immune cell infiltration levels can vary considerably between different tumour types and disease stages. For instance, tumours such as melanoma and non‐small cell lung cancer typically exhibit robust T cell infiltration, while others, including ovarian and prostate cancers, often show a relatively weaker immune presence. This disparity may explain why some tumours are more responsive to immunotherapies than others. Increased infiltration of CD8+ T cells is often associated with favourable prognosis, whereas higher levels of regulatory T cells (Tregs) tend to suppress immune responses and may contribute to reduced therapeutic efficacy.

The application of scRNA‐seq has been pivotal in elucidating these immune dynamics by providing high‐resolution, comprehensive profiles of tumour‐infiltrating immune cells [19]. In studies of breast cancer, for example, T cells, B cells, and macrophages were identified as the predominant immune cell types within the tumour. Notably, many of the T cells exhibited signs of exhaustion, which likely impairs their anti‐tumour efficacy. Concurrently, macrophages often demonstrated an M2‐like phenotype, which is generally linked to immune suppression and tumour promotion [35]. These findings highlight the critical role of immunosuppressive cell populations in shaping tumour biology and suggest potential therapeutic targets. The high sensitivity and resolution of scRNA‐seq enable precise classification of immune cells based on their gene expression profiles, allowing researchers to identify distinct T cell subtypes and analyse their functional states more effectively. In the context of colorectal cancer, large‐scale single‐cell profiling of T cells has been employed to investigate their distribution, clonal expansion, and differentiation pathways. By mapping specific gene expression signatures and T cell receptor profiles, researchers can trace immune cell dynamics within the tumour microenvironment. This detailed characterisation of immune cell behaviour paves the way for the development of more targeted and personalised immunotherapeutic strategies, offering hope for improved clinical outcomes in cancers such as ovarian cancer [6].

## 17 Tracking Immune Therapy Effects With scRNA‐Seq

What makes scRNA‐seq so useful is that it allows us to see immune cells in their native environment, rather than just looking at an average of what's happening in the whole tumour. It can detect rare cell types and identify gene expression patterns that suggest how those cells might be interacting with the cancer. This level of detail is critical for understanding the immune landscape in ovarian cancer and its heterogeneity. For example, scRNA‐seq helps researchers figure out how different immune cells talk to each other and whether they're helping or hurting the tumour. This is something that wasn't possible before because traditional methods only looked at large tumour samples and missed a lot of the nuance [33].

More specifically, scRNA‐seq can identify which pathways are active in immune cells within the tumour [19]. By comparing the profiles of immune cells that are found in more aggressive or resistant tumours to those in less aggressive ones, researchers can begin to spot patterns that might be important for treatment. It may also help pinpoint certain immune cells or signalling pathways that could be targeted by new therapies. For example, therapies that boost the activity of cytotoxic T cells or block immunosuppressive pathways may hold promise for ovarian cancer patients with specific immune profiles [38]. scRNA‐seq seems to be quite useful for monitoring how immune‐targeted therapies reshape the tumour environment. In studies of anti‐CD47 treatment, single‐cell data showed an increase in CD4+ and CD8+ T cells, along with a decrease in regulatory T cells. This shift may indicate a more active immune response against the tumour. At the same time, there was a reduction in immunosuppressive macrophages, which likely contributes to improved anti‐tumour activity [37]. These kinds of changes are hard to capture with bulk sequencing, where signals from different cells get averaged out [19]. In contrast, scRNA‐seq provides a more detailed view, making it easier to detect subtle but potentially important immune shifts. scRNA‐seq allows for a more precise characterisation of immune cell populations in tumours. It helps reveal not just which cells are present, but also their functional states and interactions. This level of detail may contribute to understanding why some tumours resist immunotherapy while others respond. It also helps identify specific immune cell subsets that could be targeted in treatment. As immunotherapy continues to develop, scRNA‐seq will likely remain an important tool for improving how these therapies are designed and applied across different cancer types [12].

## 18 Intercellular Communication in Tumours

Tumours are not just clusters of cancer cells, but rather complex ecosystems made up of malignant, immune, and stromal components. The composition of these cells, and how they interact, seems to play a major role in tumour progression. Communication between these cells occurs through several mechanisms, including ligand‐receptor binding and different forms of signalling like paracrine and autocrine pathways. These interactions may influence cell behaviour in multiple ways, such as altering growth rates, invasion capacity, and metastatic potential. As a result, the tumour microenvironment is constantly being reshaped by these ongoing cellular exchanges. There has been a shift in recent years away from focusing only on tumour cells toward a broader view that includes the surrounding microenvironment. Tumour cells do not act independently; instead, they continuously interact with nearby stromal and immune cells. scRNA‐seq makes it possible to study these interactions in detail by profiling different cell populations and examining their communication networks. It also allows comparisons across patients and conditions, which may help predict disease progression or treatment response. This kind of approach seems especially relevant for developing more targeted therapies [12].

Research into tumour heterogeneity suggests that communication between cancer cells and stromal cells is a key driver of disease progression. Tumour cells can release signalling molecules like interleukins, which recruit immune cells such as macrophages. They can also produce factors like VEGF that promote blood vessel formation, supporting tumour growth. scRNA‐seq studies have mapped large numbers of ligand‐receptor interactions across different cell types, revealing dense communication networks. These networks often involve chemokines, growth factors, and extracellular matrix components, all of which may contribute to tumour expansion and immune modulation. Similar patterns have been observed in glioma, where scRNA‐seq has been used to study interactions between tumour‐associated macrophages and stem‐like tumour cells. Certain ligand‐receptor pairs, such as Notch‐related signals and other growth‐related factors, appear to be highly active in these settings. These interactions may influence processes like cell proliferation, invasion, and structural organisation. Some studies have even used these signalling patterns to build prognostic models, suggesting that intercellular communication is not just biologically important but also clinically relevant [35].

## 19 Modelling Cell–Cell Communication in Tumours

In one study, computational modelling was applied to scRNA‐seq datasets to explore how cells within tumours communicate with each other. The approach seems to rely on identifying ligand‐receptor pairs across single‐cell profiles, which allows for a more systematic mapping of signalling networks. Researchers tested this framework in several murine tumour models, covering six tumour types, and then extended it to human metastatic melanoma samples. Interestingly, chemokine‐related interactions, particularly those involving CCR1, CCR2, and CCR5, showed the strongest signals. This pattern may suggest that chemokine signalling plays a dominant role in coordinating interactions across different cell populations [39]. Macrophages, in particular, appeared to sit at the centre of these networks, likely influencing both immune responses and structural features of the tumour microenvironment. Taken together, these findings reinforce the idea that scRNA‐seq is not just descriptive but also quite useful for inferring functional relationships between cells. It enables the identification of signalling hubs and highlights which cell types may be disproportionately influential. This kind of systems‐level perspective seems to move beyond simply cataloguing cell types toward understanding how tumours behave as dynamic ecosystems. In that sense, scRNA‐seq may contribute to more targeted therapeutic strategies, especially those that aim to disrupt communication pathways rather than just eliminate tumour cells. It also raises the possibility that targeting non‐malignant cells within the tumour could be just as important. The technology continues to reshape how tumour biology is conceptualised [12].

## 20 Immune and Stromal Complexity in Ovarian Tumours

Within the ovarian cancer microenvironment, immune‐infiltrating cells appear to play somewhat contradictory roles. On one hand, they contribute to immune surveillance, with natural killer cells and cytotoxic T lymphocytes exerting antitumor effects. On the other hand, these same populations or closely related ones can promote tumour progression under certain conditions. This duality seems especially evident in tumour‐associated macrophages and neutrophils, which release factors like interleukins, TNF, and VEGF. These molecules may enhance angiogenesis and support tumour growth, creating a microenvironment that favours disease progression. So, immune cells are not simply protective; their function likely depends heavily on context and signalling cues [40]. More recently, attention has shifted toward how these immune populations influence responses to immunotherapy. Their functional plasticity makes them attractive, though complex, therapeutic targets. For example, their interactions with checkpoint pathways or engineered T cells may either enhance or limit treatment efficacy. The variability in their phenotypes seems to arise from ongoing communication with tumour and stromal cells, which further complicates their classification. This suggests that understanding immune cell behaviour requires looking at the broader network rather than isolated cell types. As a result, the tumour microenvironment is increasingly viewed as a highly interactive system rather than a static composition of cells [11] (Table 3).

**TABLE 3:** Key findings in ovarian cancer heterogeneity and immune response [41, 42].

Feature	Description	Implications for treatment	Challenges
Malignant cell subpopulations	Subtypes with distinct transcriptional profiles, including stem‐like and EMT‐like populations.	Associated with poor prognosis and resistance to chemotherapy.	Difficulty in targeting specific subpopulations effectively.
Immune‐infiltrating cells (IICs)	Includes T cells, macrophages, and dendritic cells.	Immunosuppressive roles of macrophages and Tregs complicate treatment.	Immune cell functional diversity and context‐dependent roles.
Metastasis and microenvironment	Changes in omental metastasis due to lipid‐rich environment.	Reveals new pathways for targeting metastasis in ovarian cancer.	Tumour evolution is non‐linear and dynamic.
T‐cell exhaustion	High levels of exhausted T cells linked to treatment resistance.	Targeting exhausted T cells may improve therapeutic outcomes.	Understanding the transition from active to exhausted states.
Tumour‐stroma interaction	Tumour cells interact with immune and stromal cells through ligand‐receptor binding.	Disrupting these interactions may provide new therapeutic strategies.	Targeting stromal and immune cells requires overcoming complexity.

## 21 Insights From Single‐Cell Profiling

Single‐cell RNA sequencing has made it possible to dissect this complexity in much finer detail. Early work identified several key immune populations in ovarian tumours, including macrophages, T cell subsets, and dendritic cells, each with distinct transcriptional profiles. These differences likely reflect both functional specialisation and environmental conditioning. In addition to immune cells, cancer‐associated fibroblasts have also emerged as important regulators of immune activity. Their presence seems to influence how immune cells are recruited and activated within the tumour [43]. As more studies incorporate these nonimmune components, the overall picture becomes increasingly intricate. Macrophages, in particular, stand out due to their diversity and functional range. Tumour‐infiltrating macrophages and tumour‐associated macrophages both originate from circulating monocytes, yet they seem to diverge in behaviour once the tumour. Tumour‐infiltrating macrophages may represent more recently recruited cells that are still responsive to environmental cues. In contrast, tumour‐associated macrophages often adopt a more stable phenotype that supports tumour growth and immune suppression. While there is some overlap between these groups, their regulatory roles are not identical. This distinction, although somewhat fluid, may be important for understanding disease progression [44].

## 22 Beyond the M1/M2 Framework

The classical M1/M2 polarisation model is still widely used but appears increasingly insufficient. M1 macrophages are typically described as pro‐inflammatory and tumour suppressive, whereas M2 macrophages are linked to tumour‐promoting activities. scRNA‐seq data suggest that macrophage states exist along a spectrum rather than in discrete categories. Quantitative analyses have attempted to capture this by estimating M1/M2 ratios, which may carry prognostic value. In ovarian cancer, a predominance of M2‐like macrophages has been associated with poorer outcomes and resistance to treatments such as cisplatin. Certain genes, including SLAMF7 and GNAS, seem to be involved in this resistance, though the mechanisms are still being clarified [45]. Autophagy also appears to play a role in defining macrophage behaviour. M2‐like macrophages with high autophagic activity have been linked to increased chemoresistance, suggesting a possible therapeutic angle. In metastatic sites like the omentum, immune cell clusters form structures that resemble lymphoid aggregates. These areas seem to attract tumour cells, creating a distinct microenvironment that differs from the primary tumour. Studies using single‐cell approaches have shown that macrophage activation states vary between these sites. Even macrophages that appear M1‐like may express atypical markers, indicating hybrid or transitional states. This further complicates the traditional classification system [33].

## 23 Macrophage Diversity and Plasticity

Recent single‐cell studies in high‐grade serous ovarian cancer have identified a surprisingly large number of macrophage subpopulations—at least ten in some analyses. Early‐stage macrophages tend to exhibit more immunostimulatory features, while more differentiated subsets show gene expression patterns associated with tumour support. These later‐stage cells may also be less effective at recruiting other immune cells, which could contribute to immune evasion. The relative abundance of these subtypes seems to correlate with patient outcomes, suggesting that macrophage composition is clinically relevant. This level of detail would likely not have been captured without single‐cell approaches [46]. A defining feature of macrophages is their plasticity, which allows them to shift phenotypes in response to environmental signals. Cytokines such as CCL2, CSF‐1, and TNF‐α appear to drive these transitions, along with metabolic conditions in the tumour. Non‐coding RNAs may also regulate these changes, adding another layer of complexity. Some macrophages express receptors like C5aR1, which seem to suppress T cell activity and promote angiogenesis. Additionally, signalling interactions involving IL‐6 and oncostatin M may enhance tumour invasiveness through pathways like STAT3. These interconnected processes highlight how macrophages can actively shape tumour behaviour, rather than simply responding to it [47].

Tumour‐Infiltrating Macrophages (TIMs) and Tumour‐Associated Macrophages (TAMs) are two terms often used interchangeably to describe macrophages within the tumour microenvironment, but they refer to different populations with distinct functional roles and origins. TIMs specifically refer to macrophages that are recruited into the tumour site from the circulation. These cells typically exhibit a dynamic functional profile, which can be influenced by the local tumour environment. TIMs are often associated with the inflammatory response and may display either pro‐inflammatory or anti‐inflammatory properties depending on signals from the tumour microenvironment [48]. On the other hand, TAMs are macrophages that reside within the tumour tissue and can arise from different sources, including local tissue‐resident macrophages or monocytes that have infiltrated the tumour. TAMs generally adopt an immunosuppressive phenotype, promoting tumour growth, angiogenesis, and immune evasion, often by secreting cytokines such as IL‐10 and TGF‐β. The functional plasticity of both TIMs and TAMs is heavily influenced by the tumour's biological context, with TIMs often switching their functional state in response to specific tumour‐derived factors. Understanding the distinction between these two macrophage populations is crucial for developing targeted therapies aimed at reprogramming these cells to enhance anti‐tumour immunity [11].

## 24 Closing Thoughts on Cellular Complexity in OC

Single‐cell approaches have really shifted how we look at the ovarian cancer tumour microenvironment, especially when it comes to teasing apart immune and stromal cell behaviour. What comes through quite clearly is that this ecosystem is not only diverse but also constantly changing in ways that are not always predictable. Macrophages, in particular, seem to sit at the centre of this complexity, partly because of how flexible their phenotypes are. They can switch roles depending on context, which likely contributes to tumour progression as well as immune suppression. At the same time, their regulatory influence may shape how other immune cells behave, sometimes in ways that blunt therapeutic responses. So, while current treatments target broad mechanisms, it seems increasingly necessary to refine strategies based on these nuanced cellular interactions. Continued work in this area will probably help identify more precise intervention points [2].

## 25 T Cells in the Tumour Microenvironment

T cells play a central but somewhat complicated role in the ovarian cancer microenvironment. Not all subsets are equally effective at killing tumour cells, even though T cell‐based therapies are still a major focus in oncology. In ovarian cancer, tumour‐infiltrating lymphocytes are mostly made up of CD8+ cytotoxic cells, CD4+ helper cells, and regulatory T cells. There is a general trend where higher overall T‐cell infiltration, combined with fewer regulatory T cells, corresponds to better outcomes. Still, this broad classification does not fully explain why many patients do not respond well to immunotherapy. It suggests that simply counting T cells is not enough; their functional state likely matters more than their abundance [49]. This idea has been gaining traction but is still not fully resolved. One possible explanation involves the suppressive environment shaped by other immune cells, especially macrophages. These cells may interfere with T‐cell activity, limiting their ability to effectively kill tumour cells. In addition, a notable portion of CD8+ T cells appears to be non‐reactive to the patient's own tumour, which raises questions about antigen recognition. This implies that only a subset of T cells is actually contributing to anti‐tumour immunity. Identifying these functional subsets seems critical if therapies are to become more targeted and effective. Without that distinction, treatments may continue to show inconsistent results [50].

Some studies have tried to define these effective T‐cell populations more precisely using surface markers. For instance, certain CD8+ T cells that co‐express markers like CD39, CD103, and PD‐1 appear to have stronger cytotoxic potential and better tumour specificity. These cells also seem to correlate with improved clinical outcomes, although the mechanisms are still being worked out. In parallel, patterns of immune cell infiltration have been used to classify tumours into subtypes with distinct immune landscapes. Tumours with high infiltration tend to show specific T‐cell phenotypes, alongside other immune components like plasma cells and plasmablasts. This layered composition suggests that the immune response is coordinated across multiple cell types rather than driven by T cells alone. It may also explain variability in patient responses [51].

## 26 T‐Cell Diversity and Functional States

Looking more closely, T‐cell populations in ovarian cancer are highly heterogeneous. Single‐cell RNA sequencing studies have identified multiple CD8+ T‐cell subgroups, each with distinct transcriptional profiles. Among these, tissue resident memory T cells seem particularly enriched within tumour tissue. These cells are generally associated with localised immune defence, but their long‐term behaviour in tumours is less clear. At the same time, a significant fraction of CD8+ T cells expresses markers associated with exhaustion. These exhausted cells may originate from earlier, more active states, gradually losing functionality as the tumour develops [52]. This transition could be a key factor in why immune responses weaken over time. Interestingly, some of the signalling molecules produced by these T cells, such as interleukins and NOTCH ligands, may have dual roles. While they support immune activation initially, they might also contribute to eventual exhaustion under chronic stimulation. There are also indications that other pathways, including those involving immune checkpoint molecules, play a role in maintaining this suppressed state. These overlapping mechanisms make it difficult to pinpoint a single driver of dysfunction. Instead, it seems more likely that exhaustion arises from a combination of persistent antigen exposure and environmental signals within the tumour [53].

Interactions between macrophages and T cells add another layer of complexity. Macrophages can release chemokines that attract certain T‐cell subsets, including tissue‐resident memory cells. However, this recruitment does not necessarily lead to effective tumour killing. In some cases, T cells may become localised in regions where their cytotoxic activity is limited, which could inadvertently support immune evasion. This spatial aspect of immune regulation is still being explored but seems quite important. Altogether, these dynamics suggest that improving T cell–based therapies will require not just enhancing T‐cell activity, but also modifying the surrounding microenvironment to support their function [54].

## 27 Dendritic Cells in the OC Microenvironment

Dendritic cells seem to occupy a somewhat contradictory position in the ovarian cancer microenvironment. On one hand, they are essential for antigen presentation and can activate T cells in a way that supports anti‐tumour immunity. On the other hand, signals from the tumour environment often appear to shift their behaviour toward a more suppressive role. The surrounding milieu, which is typically rich in cytokines like TGF‐β, IL‐10, and CXCL‐12, likely plays a part in this shift. These factors seem to attract certain precursor populations, particularly plasmacytoid dendritic cells. Once present, these cells may start producing IL‐10, which can dampen cytotoxic T‐cell responses. So instead of activating immunity, they may end up reinforcing suppression [55]. There is also evidence that dendritic cells derived from the bone marrow adopt an immunosuppressive phenotype in this setting. These cells often show increased expression of molecules such as PD‐L1 and Fgl2, both of which are associated with reduced T‐cell activity. This shift seems to promote the expansion of regulatory T cells while limiting T‐cell proliferation. As a result, the tumour microenvironment becomes more tolerant to cancer growth. Similar dendritic cell populations have been identified in ascitic fluid, especially in patients with more advanced or metastatic disease. These cells tend to remain in an immature state and may also contribute to angiogenesis, which further supports tumour progression. They are often grouped under the term tumour‐associated dendritic cells, though that category likely includes a mix of functional states [56].

More recent work suggests that not all dendritic cells in this environment are suppressive. A subset known as monocyte‐derived dendritic cells has been identified, particularly within ascites. These cells appear capable of promoting the differentiation of cytotoxic CD8+ T cells, which points to a more immunogenic role. Their presence complicates the picture but also opens up some interesting possibilities. It seems plausible that boosting the activity of these specific dendritic cell subsets could improve immune responses against the tumour. In particular, enhancing their ability to cross‐present antigens might be useful for immunotherapy strategies. Still, how to selectively target or expand these beneficial populations remains an open question [57].

## 28 Emerging Clinical Directions in OC

Current immunotherapy approaches in ovarian cancer still face some pretty clear limitations, mostly due to the strongly immunosuppressive nature of the tumour microenvironment. This suppression seems to be driven by several cell types at once, including regulatory T cells, tumour‐associated macrophages, and cancer‐associated fibroblasts, along with the gradual exhaustion of cytotoxic T cells. Together, these factors likely reduce the overall effectiveness of immune‐based treatments. Because of this, one practical direction has been to try and limit or reprogram these inhibitory components rather than only boosting immune activation [58]. For example, strategies targeting macrophages often focus on blocking their recruitment or shifting them toward a more pro‐inflammatory state. These approaches are still being refined, but they seem to offer a more balanced way of restoring immune function within tumours. Single‐cell sequencing has become an important tool in this context, mainly because it allows researchers to look at the tumour environment at much higher resolution [59]. Instead of averaging signals across many cells, it reveals distinct subpopulations and their interactions, which may otherwise go unnoticed. This has helped identify more precise therapeutic targets, especially within immune and stromal compartments. In some cases, it has also supported the development of dendritic cell–based approaches, where patient‐specific immune cells are modified to improve antigen presentation. These kinds of strategies are still somewhat experimental, but they reflect a broader shift toward more tailored immunotherapy. It seems likely that future treatments will rely more heavily on this level of cellular detail [60].

**FIGURE 3:**
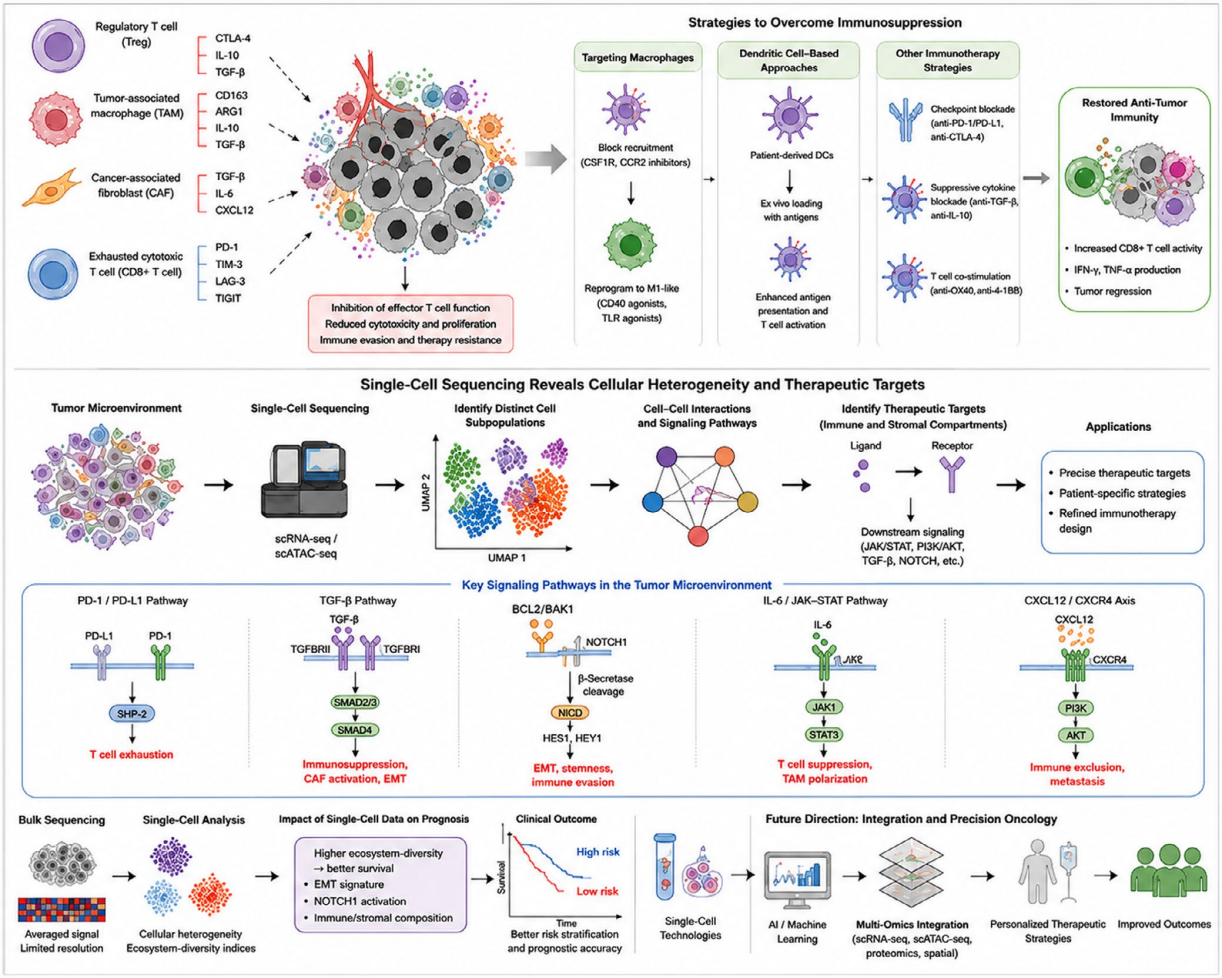
Schematic representation of current immunotherapy challenges and strategies in ovarian cancer. This diagram illustrates the immunosuppressive tumour microenvironment, highlighting the roles of regulatory T cells, tumour‐associated macrophages, cancer‐associated fibroblasts, and exhausted cytotoxic T cells in limiting immune‐based therapies. It also depicts potential therapeutic approaches targeting these inhibitory components, such as macrophage reprogramming and dendritic cell‐based strategies. Single‐cell sequencing is shown as a key tool for identifying distinct tumour subpopulations and guiding personalised treatments.

Beyond therapy, single‐cell data is also starting to reshape how prognosis is assessed in ovarian cancer. Traditional bulk sequencing methods are being supplemented, and sometimes replaced, by metrics that capture cellular diversity within tumours. Measures like ecosystem diversity or similar indices appear to provide a more nuanced view of tumour composition. This may help explain differences in patient outcomes that are not obvious from standard classifications. In addition, processes like epithelial‐to‐mesenchymal transition, along with signalling pathways such as NOTCH1, have been linked to prognosis using these approaches [61]. These connections are still being explored, but they seem to add another layer of clinical relevance. Looking ahead, the integration of single‐cell technologies with computational methods like artificial intelligence and machine learning could push this field further. These tools are particularly useful for handling large and complex datasets, which are typical in single‐cell studies. They may improve how patterns are detected and how treatment strategies are designed on an individual level. There is also some expectation that these approaches could support earlier detection or more precise intervention strategies. While this area is still evolving, it likely represents a key direction for personalised oncology. Combining biological insight with computational modelling may significantly change how ovarian cancer is managed [62] (Figure 3).

## 29 Rethinking the Tumour Microenvironment

Over the past few decades, the way cancer biology is understood has shifted quite a bit. Earlier work focused mostly on genetic mutations within tumour cells themselves, but that view now seems somewhat incomplete. The tumour microenvironment is increasingly seen as a dynamic and interactive system, not just a passive background. It includes a range of immune and stromal cells, such as T cells, macrophages, B cells, NK cells, fibroblasts, and dendritic cells. These populations do not act in isolation; instead, they constantly interact in ways that likely shape both tumour progression and treatment response. This more integrated perspective seems to better explain why tumours behave so differently across patients. It also suggests that targeting the environment, not just the tumour, may be necessary [63].

The hope is that by understanding the immune landscape at such a granular level, doctors can one day predict which therapies will work best for each patient. For example, we might be able to tell if a patient's tumour is “hot” (meaning it's filled with immune cells that are actively attacking the tumour) or “cold” (with few immune cells). This could help doctors decide whether a patient would benefit from treatments like immune checkpoint inhibitors, which are designed to boost the immune response against the tumour [3].

## 30 Limits of Current Tools and What Single‐Cell Adds

Traditional experimental approaches have struggled to fully capture this level of complexity. Most bulk methods tend to average signals across many cells, which can obscure important differences between subpopulations. Single‐cell sequencing, in contrast, allows for much finer resolution and has started to reveal distinct cellular phenotypes and functional states within tumours [64]. In ovarian cancer, this has been particularly useful for studying stromal components and their role in immune regulation. Still, the use of these technologies remains somewhat limited. Certain cell types, like neutrophils, are often underrepresented, possibly due to technical issues such as low RNA quality or difficulties in sample preparation. On top of that, the cost of single‐cell approaches can be a barrier, especially when compared to other methods like spatial transcriptomics. So, while promising, the approach is not yet widely accessible [65]. Although single‐cell RNA sequencing is a valuable and less expensive alternative to some techniques, the high cost of scRNA‐seq compared to bulk RNA‐seq remains a limitation for broader adoption. Additionally, spatial transcriptomics, while providing complementary spatial resolution, can be even more costly [66].

## 31 Methodological Gaps and Future Integration

There are also some clear gaps in how single‐cell data is currently analysed and integrated. Existing workflows often fall short when it comes to combining different layers of biological information. Approaches that integrate multiple data types, such as chromatin accessibility, protein expression, and antigen specificity, are starting to address this but are still developing [67]. Techniques like scATAC‐seq, CITE‐seq, and similar platforms seem to offer deeper insights, though they also add complexity. At the same time, computational methods, especially those based on machine learning, are becoming more important for handling these large datasets. These tools may help uncover patterns that are not obvious through standard analysis. If combined effectively, these approaches could improve both biological understanding and clinical application [15].

In the future, scRNA‐seq could guide the use of combination therapies. If researchers can identify key immune pathways that are blocked in certain ovarian tumours, they might be able to develop therapies that target those blocks, potentially restoring the immune system's ability to fight the cancer. This is especially critical because ovarian cancer often develops resistance to treatment, and finding new ways to restore immune function could be key to overcoming that resistance [68].

## 32 Conclusion and Final Perspective

scRNA‐seq is helping to peel back the layers of complexity in ovarian cancer. It provides a detailed look at the tumour's immune landscape and gives us new tools to understand how tumours adapt and fight back against treatments [31]. With this technology, we're moving closer to more personalised, effective therapies for ovarian cancer that could improve survival and reduce the challenges posed by tumour heterogeneity. However, more work is needed to translate these findings into clinical practice, particularly when it comes to validating biomarkers and identifying the best therapeutic strategies for different patient profiles [13]. Single‐cell sequencing allows for a more detailed mapping of the tumour microenvironment and may help identify new therapeutic targets that were previously overlooked. There is also potential for improving how patient outcomes are predicted, particularly by linking cellular composition to clinical behaviour. That said, the field is still working through technical and practical limitations, so its full impact is not yet realised. As methods become more refined and accessible, integration into clinical research will likely become more common. This could eventually support more personalised and effective treatment strategies, though that transition may take time.

## Author Contributions

**Sihan Chen:** writing – review, software, conceptualization, methodology. **Manoj Kumar Mishra:** data extractions, methodology, writing – review and editing. **Helen Cai:** supervision, validation, writing – review and editing. **Usamah Sayed:** writing – review and editing, visualisation. **Wedad M. Alawad:** writing – review and editing, conceptualisation. **Ghada Moh. Samir Elhessewi:** writing – review and editing, methodology.

## Funding

Princess Nourah bint Abdulrahman University Researchers Supporting Project number (PNURSP2026R809), Princess Nourah bint Abdulrahman University, Riyadh, Saudi Arabia.

## Ethics Statement

The authors have nothing to report.

## Consent

The authors have nothing to report.

## Conflicts of Interest

The authors declare no conflicts of interest.

## Data Availability

Data sharing not applicable to this article as no datasets were generated or analysed during the current study.
